# Vasculogenic mimicry correlates to presenting symptoms and mortality in uveal melanoma

**DOI:** 10.1007/s00432-021-03851-9

**Published:** 2021-11-13

**Authors:** Shiva Sabazade, Viktor Gill, Christina Herrspiegel, Gustav Stålhammar

**Affiliations:** 1 St. Erik Eye Hospital, Stockholm, Sweden; 2 Department of Clinical Neuroscience, Karolinska Institutet, Eugeniavägen 12, 17164 Stockholm, Sweden; 3 Department of Pathology, Västmanland Hospital Västerås, Västerås, Sweden

**Keywords:** Uveal melanoma, Pathology, Oncology, Vasculogenic mimicry, PAS density, Prognosis

## Abstract

**Purpose:**

Fluid-conducting extracellular matrix patterns known as vasculogenic mimicry (VM) have been associated with poor prognosis in uveal melanoma and other cancers. We investigate the correlations between VM, presenting symptoms, mortality, and the area density of periodic acid-Schiff positive histological patterns (PAS density).

**Methods:**

Sixty-nine patients that underwent enucleation for uveal melanoma between 2000 and 2007 were included. Clinicopathological parameters presenting symptoms and outcomes were collected. Histological tumor sections were evaluated for VM and PAS density was quantified with digital image analysis.

**Results:**

Thirty-four patients (49%) presented with blurred vision. 18 (26%) with a shadow in the visual field, 7 (10%) with photopsia and/or floaters, and 2 (3%) with metamorphopsia. Nine patients (13%) had no symptoms at all. Median follow-up was 16.7 years (SD 2.6). A shadow in the visual field, but no other symptom, was positively correlated with the presence of VM (*φ* 0.70, *p* < 0.001) and greater PAS density (*p* < 0.001). In multivariate regression, retinal detachment (RD), presence of VM, and PAS density ≥ median were independent predictors of a shadow, but not tumor distance to the macula, tumor apical thickness, tumor diameter, or ciliary body engagement. The presence of VM was associated with significantly shorter cumulative disease-specific survival (Wilcoxon *p* = 0.04), but not PAS density ≥ median, presenting symptoms or RD (*p* > 0.28).

**Conclusion:**

Tumors from uveal melanoma patients that report a visual field shadow are likely to display VM and greater PAS density, likely explaining the previously reported association between this symptom and poor prognosis.

## Background

Uveal melanoma is the most common malignant intraocular tumor in adults. At the time of diagnosis, about 2% of patients have detectable metastases (Garg et al. 2021). However, within 15 years, between one-quarter and almost half all patients will succumb to metastatic disease with similar survival after eye-conserving plaque brachytherapy and surgical enucleation of medium sized tumors (Kujala et al. 2003; COMS 2006; Stalhammar 2020). This has been attributed to early seeding of micrometastases from the eye to distant organs, primarily the liver (Singh 2001; Callejo et al. 2007; Uner et al. 2021). Once these seeded clusters of dormant tumor cells grow into larger, radiologically detectable, macrometastases, median patient survival is a year or less (Khoja et al. 2019; Rantala et al. 2019).

Two of the strongest prognostic factors for the risk of developing macrometastases are the presence of periodic acid-Schiff (PAS) positive extracellular matrix patterns forming fluid-conducting channels (vasculogenic mimicry, VM), and the presence of a dense network of microvessels (Folberg et al. 1992, 1996; Foss et al. 1996; Mäkitie et al. 1999; Folberg and Maniotis 2004; Cao et al. 2013; Stålhammar et al. 2019).

**Fig. 1:**
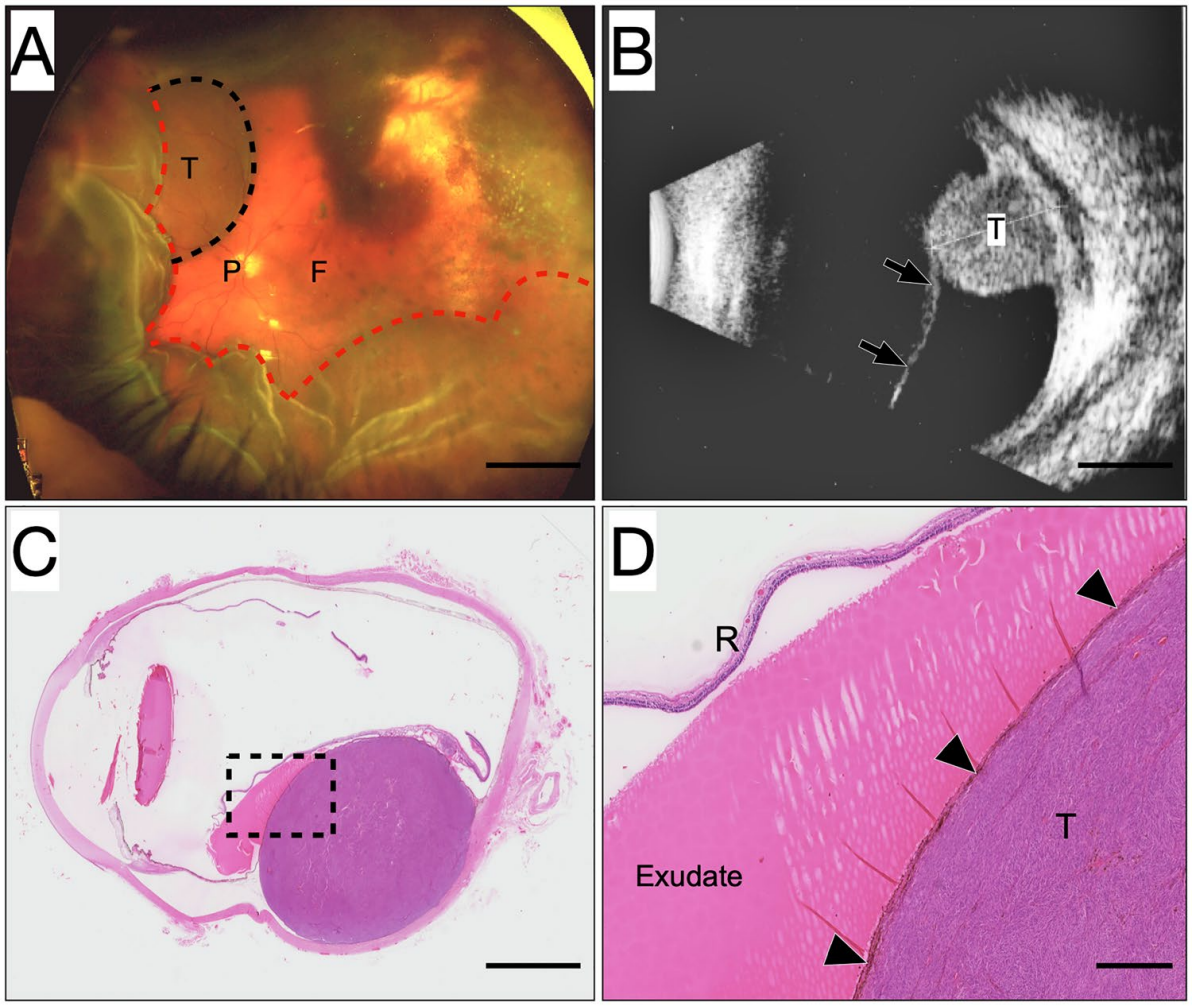
Examples of tumor-associated exudative retinal detachment. **A** Appearance in wide-field fundus photography (Optos, Inc, Dunfermline, UK). A pigmented uveal melanoma (T, outlined by the black dotted line) is situated superionasally to the optic disc (P) and fovea (F). Partially pigmented inactive tumor remnants surrounding a yellow choroidal atrophy associated with previous plaque brachytherapy are seen superiotemporally, indicating that the present tumor was a relapse outside of the treated area. Much of the nasal, temporal, and inferior retina have detached in an exudative retinal detachment by fluid leaking from the tumor (outlined by the red dotted line). **B** In ultrasonography, the detached retina (arrows) can be seen extending inferiorly from the tumor (T) apex. **C** Histological appearance. Tissue section stained with hematoxylin and eosin and digitally scanned at × 400 (Nano Zoomer 2.0 HT, Hamamatsu Photonics K.K., Hamamatsu, Japan). A large dome-shaped melanoma is located in the posterior aspect of the choroid. The normal shape of the sclera has been artefactually deformed. Scale bar 5 mm. **D** Magnification of the area within the dashed box in C. The serous exudate has leaked from the tumor (T) through Bruch’s membrane and the retinal pigment epithelium (arrowheads) and pushed the retina (R) away. Scale bar 1 mm

Presenting symptoms of uveal melanoma include blurred vision, photopsia, floaters, visual field defects, pain, and metamorphopsia (Damato and Damato 2012). In a recent report, we showed that a shadow in the visual field as a presenting symptom was associated with exudative retinal detachment (RD, Fig. 1) and larger tumor size, and that it was an independent predictor of uveal melanoma-related mortality, regardless of other simultaneous symptoms, tumor size, tumor location, local extent, and stage (Fili et al. (2021)). In univariate analysis, there has been conflicting reports on the prognostic significance of RD; some studies have found that it is a significant risk factor for tumor-related death, whereas others have indicated the contrary (Packer et al. 1992; Foss et al. 1997a, b). Regardless, there is little reason to believe that RD is directly linked to prognosis. Rather, it may be a marker for other tumor characteristics that are in turn more closely related to the risk for metastasis and tumor-related death. As indicated by multivariate regression analyses, the correlation may be related to tumor size and the presence of microvascular loops and networks (Kivela et al. 2001).

As a shadow in the visual field at presentation of uveal melanoma has been shown to be associated with retinal detachment as well as poor prognosis, and retinal detachment with tumor size and vascularity, then the shadow in the visual field should also be associated with tumor vascularity. In this paper, we therefore examine this correlation. Patients’ reports of their symptoms at the point of enucleation of an eye with uveal melanoma are correlated to histological findings including the presence of VM and the density of PAS-positive patterns.

## Methods

### Patients and samples

The study adhered to the tenets of the Declaration of Helsinki and the research group data security policy. Institutional Review Board approval was obtained from the Regional Ethical Review Board in Stockholm (Reference No. 2016/347–31/4, with amendment 2019-03485 approved by the Swedish Ethical Review Authority).

Seventy-five patients that had undergone enucleation between January 27th, 2000, and December 15th, 2007, were identified in our treatment registry along with retrospective data on gender, age at diagnosis, presenting symptoms, symptom duration before presentation, tumor thickness and tumor diameter, American Joint Committee on Cancer (AJCC) T-category, stage as well as dates of diagnosis, and last follow-up. At their first visit, patients had been asked about previous diseases, medications, family history, and symptoms. The attending ophthalmologist had generally not prompted the patient to confirm or deny a list of symptoms. Instead, the patient had been asked to describe his/her symptoms and their duration in own words. The ophthalmologist then recorded them as one or several of (1) blurred vision (decreased visual acuity), (2) shadow in the visual field, (3) photopsia and/or floaters, (4) metamorphopsia, (5) ocular pain, or (6) other, including photophobia, diplopia, and reduced perception of color. The tumor eye had also been examined with slit lamp biomicroscopy, ultrasonography, and fundus photography. The tumor’s apical thickness, largest basal diameter (LBD), and location in the choroid had been noted. Primary enucleation is typically performed within 4 weeks after diagnosis.

Furthermore, tumor sections from these 75 patients were retrieved from the archives of the St. Erik Ocular Pathology Laboratory. For diagnostic purposes, these had previously been stained with hematoxylin and eosin, as well as PAS without hematoxylin counterstain. Sections from heavily pigmented tumors were bleached before staining. No new tissue was collected, sectioned, stained, or otherwise processed. After initial histological assessment, six patients were excluded (*n* = 5 no or too little tumor tissue represented in section; *n* = 1 the represented tumor was fully necrotic). The tumor sections from the remaining 69 patients were then assessed for VM and scanned for digital measurement of PAS density according to a previously described method (Stålhammar et al. 2019).

### Follow-up

After diagnosis, patients underwent radiological screening for metastases either with a combination of chest X-ray and an ultrasonography of the liver, or by computed tomography (CT) of the chest and abdomen. The metastasis screening of the liver was then repeated by ultrasonography or by CT semi-annually for a 5-year period after diagnosis. After enucleation, patients were scheduled for routine follow-up at 1, 6, and 12 months. If local control had been achieved, patients were then examined annually for the rest of their lives.

### Vasculogenic mimicry and PAS density

Patterns of microvascular loops and networks were assessed in histological sections through a green narrow band-pass filter according to the method described by Folberg et al. (1992). The examining pathologist was blinded to all patient data, including symptoms and outcome. Presence of VM was defined as extracellular networks, closed loops, arcs with branching, or any combination of these. This definition replicates a distinction used in our previous publication, in which these patterns correlated strongly to PAS density, BAP-1 expression, gene expression class, and short metastasis-free survival (Stålhammar et al. 2019). Furthermore, the prognostic significance of the presence of loops, networks, and combined patterns have been verified in several publications from other laboratories (Foss et al. 1997a, b; McLean et al. 1997; Makitie et al. 1999).

**Fig. 2:**
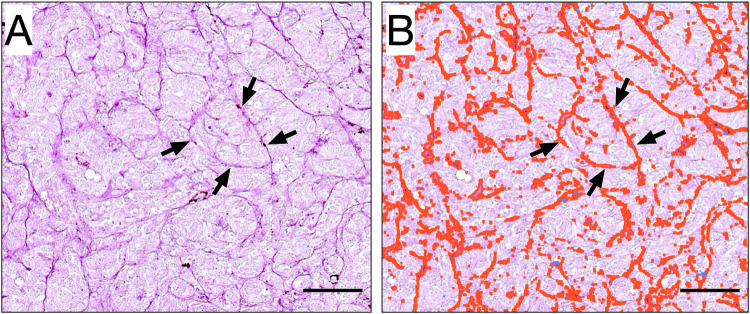
Example of vasculogenic mimicry and digital image analysis of PAS density. Tissue section stained with Periodic acid-Schiff (PAS) without hematoxylin counterstain and digitally scanned at × 400 (Nano Zoomer 2.0 HT, Hamamatsu Photonics K.K., Hamamatsu, Japan). **A** Patterns of vasculogenic mimicry can be seen, including arches (arrows). Assessments of the presence of vasculogenic mimicry were made in a light microscope with a green narrow band-pass filter (not shown in this digitally scanned section). **B** With digital image analysis of the same tumor area, the patterns have been automatically detected, highlighted and quantified. Scale bars 0.1 mm

PAS staining is mainly used for detection of structures containing a high proportion of carbohydrate macromolecules including glycogen, glycoproteins, and proteoglycans (Fu and Campbell-Thompson 2017). These are typically found in connective tissues, mucus, pericellular matrix, and basal laminae (Cerri and Sasso-Cerri 2003). Consequently, PAS will stain uveal melanoma blood vessels, but also a range of other tissue structures including vasculogenic mimicry, macrophages, and glycogen stored in the cytoplasm of tumor and non-tumor cells (Foss et al. 1997a, b; Nowak et al. 1998; Meersseman et al. 2011). The density of PAS-positive structures was evaluated with digital image analysis according to our previously described method (Stålhammar et al. 2019). In short, histological sections stained with PAS were scanned at 40 × , using a Nano Zoomer S60 (Hamamatsu Photonics K.K., Hamamatsu, Japan) at the Department of Pathology, Västmanland Hospital Västerås, Sweden. All tumor tissue in each section was then analyzed using QuPath Bioimage analysis v. 0.2.2 (Bankhead et al. 2017) for the density of blood vessels and extracellular matrix, defined as the number of PAS-positive pixels divided by the total number of pixels in the full tumor cross-section (Fig. 2). The sclera, Bruch’s membrane, retina, vitreous and tumor areas with intense inflammation, abundant pigmentation, fibrosis, bleeding, necrosis, tissue folds, and areas of poor tissue fixation were excluded from analysis.

### Statistical methods

When evaluated by the Shapiro–Wilk test, the deviation from normal distribution was not statistically significant for any of our continuous variables (*p* > 0.05) except PAS density (*p* = 0.001). We therefore used the Mann–Whitney *U* test for the latter and independent samples Student's *t* test when comparing all the other variables, with variances assumed according to Levene's test for equality of variances (equal variances assumed when *p* > 0.05). The relationship between binary variables was examined with the Phi coefficient (*φ*). For analysis of outcome in relation to presenting symptoms, the cumulative disease-specific survival was calculated. Disease-specific survival was defined as the proportion of patients not deceased from uveal melanoma. Median follow-up was defined as the median time (years) from diagnosis to the date of data collection for patients that were alive according to the national population register. Differences with a *p* < 0.05 were considered significant, all *p* values being two-sided. All statistical analyses were performed using SPSS statistics version 27 (IBM, Armonk, NY, USA).

## Results

### Descriptive statistics

A total of 69 patients with 69 tumors were included. Their mean age at diagnosis was 63 years (standard deviation, SD 12), and the mean tumor thickness and diameter 8.2 mm (SD 3.2) and 14.3 mm (SD 4.4), respectively. Most tumors (43 of 69, 62%) were of American Joint Committee on Cancer (AJCC) T-category 2 or 3. The median time elapsed from diagnosis to enucleation was 0.4 months (mean 4.6, SD 7.5). Twenty-three patients (33%) had undergone plaque brachytherapy prior to enucleation. The reason for the secondary enucleation was tumor progression or lack of regression after brachytherapy in 15, intraocular bleeding and/or elevated intraocular pressure in four, very low visual function in two, pain in one, and a scleral abscess in one. Fifty-three of 69 patients (77%) had deceased before the end of follow-up, of which 36 (52% of full cohort, 68% of deaths) died of metastasized uveal melanoma. Median follow-up for the 13 survivors was 16.7 years (SD 2.6, Table 1).

**Table 1:** Demographics and clinical features of study patients and tumors

*n* =	69
Mean age at diagnosis, years (SD)	63 (12)
Sex, *n* (%)	
Female	27 (39)
Male	43 (61)
Tumor eye laterality, *n* (%)	
Right	35 (51)
Left	34 (49)
Months from diagnosis to enucleation, median (SD)	0.4 (7.5)
Previous brachytherapy, *n* (%)	23 (33)
Mean tumor thickness, mm (SD)	8.2 (3.2)
Mean tumor diameter, mm (SD)	14.3 (4.4)
Mean tumor distance to optic disc, mm (SD)	3.2 (3.8)
AJCC T-category, *n* (%)	
1	9 (13)
2	20 (29)
3	23 (33)
4	17 (25)
AJCC stage, *n* (%)	
I	9 (13)
IIA	20 (29)
IIB	23 (33)
IIIA	17 (25)
IIIB	0 (0)
IIIC	0 (0)
IV	0 (0)
Median follow-up, years (SD, min.–max.)	16.7 y (2.6, 12.5–20.3)

*SD* standard deviation, *AJCC* American Joint Committee on Cancer

Thirty four of 69 patients (49%) presented with blurred vision. 18 (26%) with a shadow in the visual field, 7 (10%) with photopsia and/or floaters, 2 (3%) with metamorphopsia, and 0 presented with ocular pain and/or with other symptoms. Nine patients (13%) had no symptoms at all (Table 2). The mean symptom duration before diagnosis was 4 months (SD 5).

**Table 2:** Distribution of presenting symptoms

Symptom	*n* (%)
Blurred vision	34 (49)
Shadow in visual field	18 (26)
Photopsia and/or floaters	7 (10)
Metamorphopsia	2 (3)
Ocular pain	0 (0)
Other	0 (0)
No symptoms	9 (13)

### Vasculogenic mimicry

**Fig. 3:**
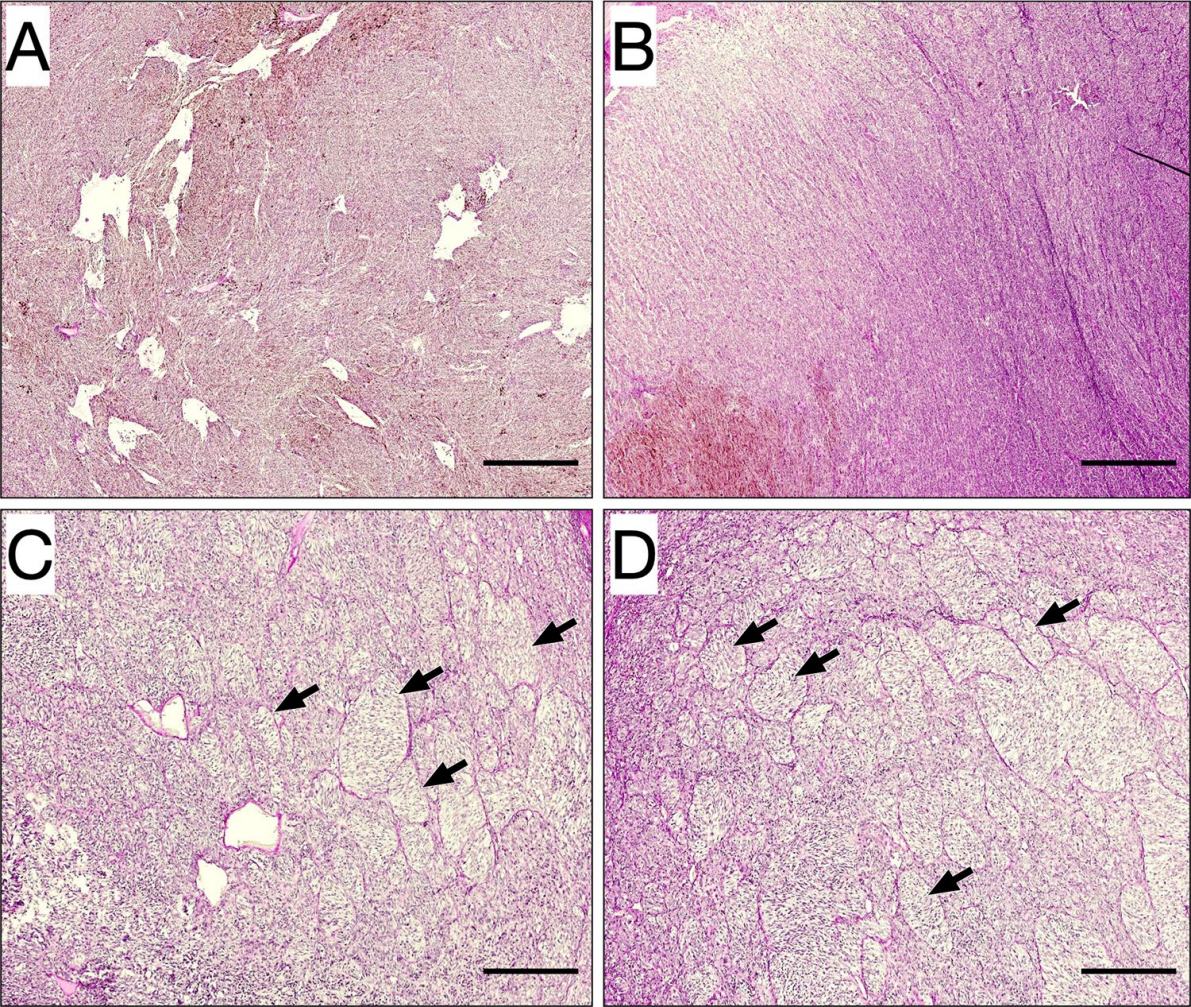
Examples of tumors with and without vasculogenic mimicry. **A** and **B** In these two tumors, no vasculogenic mimicry was identified. Tumor surface is relatively homogenous with PAS-positive patterned extracellular matrix. **C** and **D** In two other tumors, vasculogenic mimicry was identified, defined as extracellular networks, closed loops, arcs with branching, or any combination of these. Some of these patterns are indicated (arrows). Assessments of the presence of vasculogenic mimicry were made in a light microscope with a green narrow band-pass filter (not shown in this digitally scanned section). Scale bars 0.25 mm

Patterns of VM were identified in 23 of 69 tumors (33%). VM was identified in 6 of 34 patients (18%) that presented with blurred vision; in 16 of 18 patients (89%) with a shadow in the visual field; in 0 of 7 patients with photopsia and/or floaters; in 0 of 2 patients with metamorphopsia; and in 1 of 9 patients (11%) that were asymptomatic (Fig. 3). Tumors with VM did not have larger diameters than tumors without VM (15.0 and 12.8 mm, respectively, Student’s *t* test p = 0.09). Similarly, tumors with VM did not have greater apical thickness than tumors without VM (8.3 and 7.8 mm, respectively, *p* = 0.56).

The presence of VM correlated to a shadow as a presenting symptom (*φ* 0.70, *p* < 0.001, Table 3a) but not with ocular pain, metamorphopsia, or other symptoms (*p* > 0.31). Blurred vision and photopsia and/or floaters were negatively correlated (*φ* − 0.33, *p* = 0.006 and *φ* − 0.24, *p* = 0.048, respectively).

**Table 3:** Presence of vasculogenic mimicry and PAS density in relation to a shadow in the visual field as presenting symptom.

a. Shadow in the visual field vs. vasculogenic mimicry			
	Vasculogenic mimicry present	Vasculogenic mimicry not present	Total
Shadow in the visual field	16	1	17
No shadow in the visual field	5	47	52
Total	21	48	69

b.Shadow in the visual field vs. PAS density			
	PAS density ≥ median	PAS density < median	Total
Shadow in the visual field	16	1	17
No shadow in the visual field	19	33	52
Total	35	34	69

### PAS density

**Fig. 4:**
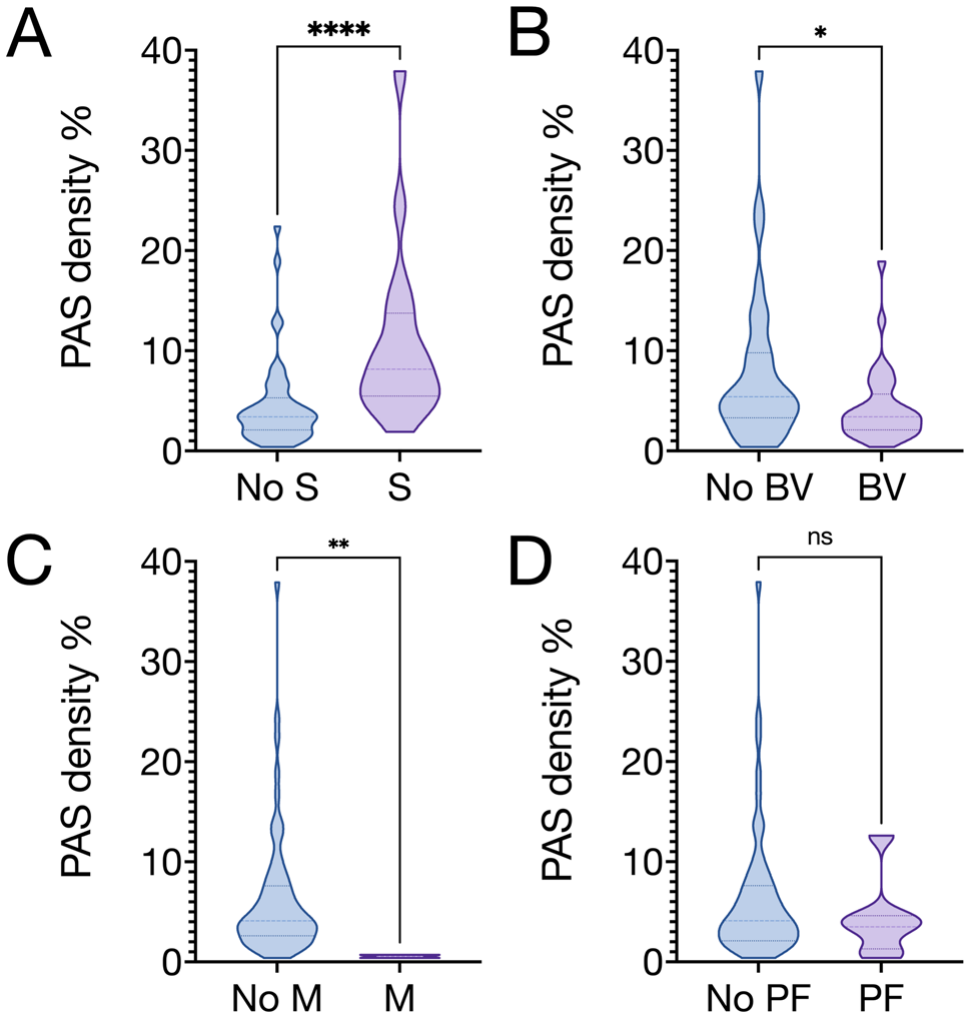
Violin plots, PAS density in relation to presenting symptoms of uveal melanoma. PAS density was defined as the number of PAS-positive pixels divided by the total number of pixels in the full tumor cross-section, as measured with digital image analysis. **A** Patients presenting with a shadow in the visual field (S) had greater PAS density than patients without a shadow (11.1 versus 4.5%, Mann–Whitney *U* *p* < 0.0001). **B** Patients presenting with blurred vision (BV) had lower PAS density than patients without blurred vision (4.4% versus 7.8%, *p* = 0.03). **C** Patients presenting with metamorphopsia (M) had lower PAS density than patients without metamorphopsia (0.5% versus 6.3%, *p* = 0.002). **D** Patients presenting with photopsia and/or floaters (PF) had similar PAS density to patients without photopsia or floaters (4.3% versus 6.3%, *p* = 0.41). *ns* non-significant on a 0.05 level. *p < 0.05. **p < 0.01. ***p < 0.001. ****p < 0.0001

The mean PAS density in all 69 tumors was 6.1% (SD 6.3, min 0.4%, max 37.9%). Patients presenting with a shadow in the visual field had greater PAS density than patients without a shadow (11.1 versus 4.5%, Mann–Whitney *U* *p* < 0.001, Fig. 4A).

On the other hand, patients presenting with blurred vision had lower PAS density than patients without blurred vision (4.4% versus 7.8%, *p* = 0.03, Fig. 4B). Similarly, patients presenting with metamorphopsia had lower PAS density than patients without metamorphopsia (0.5% versus 6.3%, *p* = 0.002, Fig. 4C).

Patients presenting with photopsia and/or floaters had similar PAS density to patients without photopsia or floaters (4.3% versus 6.3%, *p* = 0.41, Fig. 4D).

PAS density ≥ median correlated to a shadow as a presenting symptom (*φ* 0.50, *p* < 0.001, Table 3b) but not with blurred vision, ocular pain, metamorphopsia, photopsia and/or floaters, or other symptoms (*p* = 0.40–0.99). PAS density ≥ median also correlated to presence of VM (*φ* 0.55, *p* < 0.001). Tumors with PAS density ≥ median did not have larger diameters than tumors with PAS density < median (13.4 and 15.3 mm, respectively, *p* = 0.14). Similarly, tumors with PAS density ≥ median did not have greater apical thickness than tumors with PAS density < median (8.4 and 7.9 mm, respectively, *p* = 0.58).

### Shadow versus tumor histology

Patients presenting with a shadow in the visual field had tumors with similar apical thickness, diameter, distance to the optic disc, distance to the macula, AJCC T-category, and proportion of ciliary body engagement as patients that did not experience a shadow. However, the former had a significantly higher proportion of retinal detachments, and of tumors displaying vasculogenic mimicry and PAS density ≥ median (Table 4).

**Table 4:** Tumor characteristics in relation to visual field shadow

	Shadow (*n* = 18)	No shadow (*n* = 51)	p*
Mean tumor apical thickness, mm (SD)	8.1 (3.5)	7.9 (3.2)	0.86
Mean tumor diameter, mm (SD)	14.5 (4.7)	13.6 (4.4)	0.47
Mean tumor distance to optic disc, mm (SD)	4.3 (4.2)	4.5 (5.1)	0.90
Mean tumor distance to macula, mm (SD)	5.6 (4.7)	4.4 (4.3)	0.34
Mean AJCC T-category 1–4 (SD)	2.4 (0.9)	2.8 (1.0)	0.18
Ciliary body engagement, *n* (%)	3 (17)	11 (22)	0.75
Retinal detachment, *n* (%)	14 (78)	21 (41)	0.013
Vasculogenic mimicry present, *n* (%)	16 (89)	7 (14)	< 0.0001
PAS density ≥ median, *n* (%)	17 (94)	19 (37)	< 0.0001

*SD* standard deviation

*By Fisher’s exact test for categorical variables and Student’s T test for continuous variables

In a multivariate regression with all of tumor distance to the macula, tumor apical thickness, tumor diameter, retinal detachment, ciliary body engagement, presence of vasculogenic mimicry and PAS density entered as covariates, vasculogenic mimicry, and PAS density ≥ median, but none of the others were independent predictors of a shadow in the visual field. Ciliary body engagement was a negative predictor (Table 5).

**Table 5:** Multivariate regression, hazard for shadow as presenting symptom

Covariate	*B*	S.E	Wald	*p*	Exp(B)	95% CI lower	95% CI upper
Distance from macula, mm	0.3	0.2	2.6	0.11	1.3	0.9	1.8
Tumor diameter, mm	0.05	0.1	0.1	0.74	1.1	0.8	1.4
Tumor thickness, mm	− 0.2	0.2	0.4	0.53	0.9	0.5	1.4
Retinal detachment	2.5	1.4	3.1	0.08	12.3	0.7	206.1
Ciliary body engagement	− 4.9	2.2	5.1	0.02	0.007	0.0	0.5
Vasculogenic mimicry present	4.8	1.5	11.1	0.001	124.9	7.3	2149.6
PAS density	3.2	1.5	4.4	0.04	23.7	1.2	462.7
Constant	− 6.8	2.8	6.0	0.01	0.001		

### Survival

**Fig. 5:**
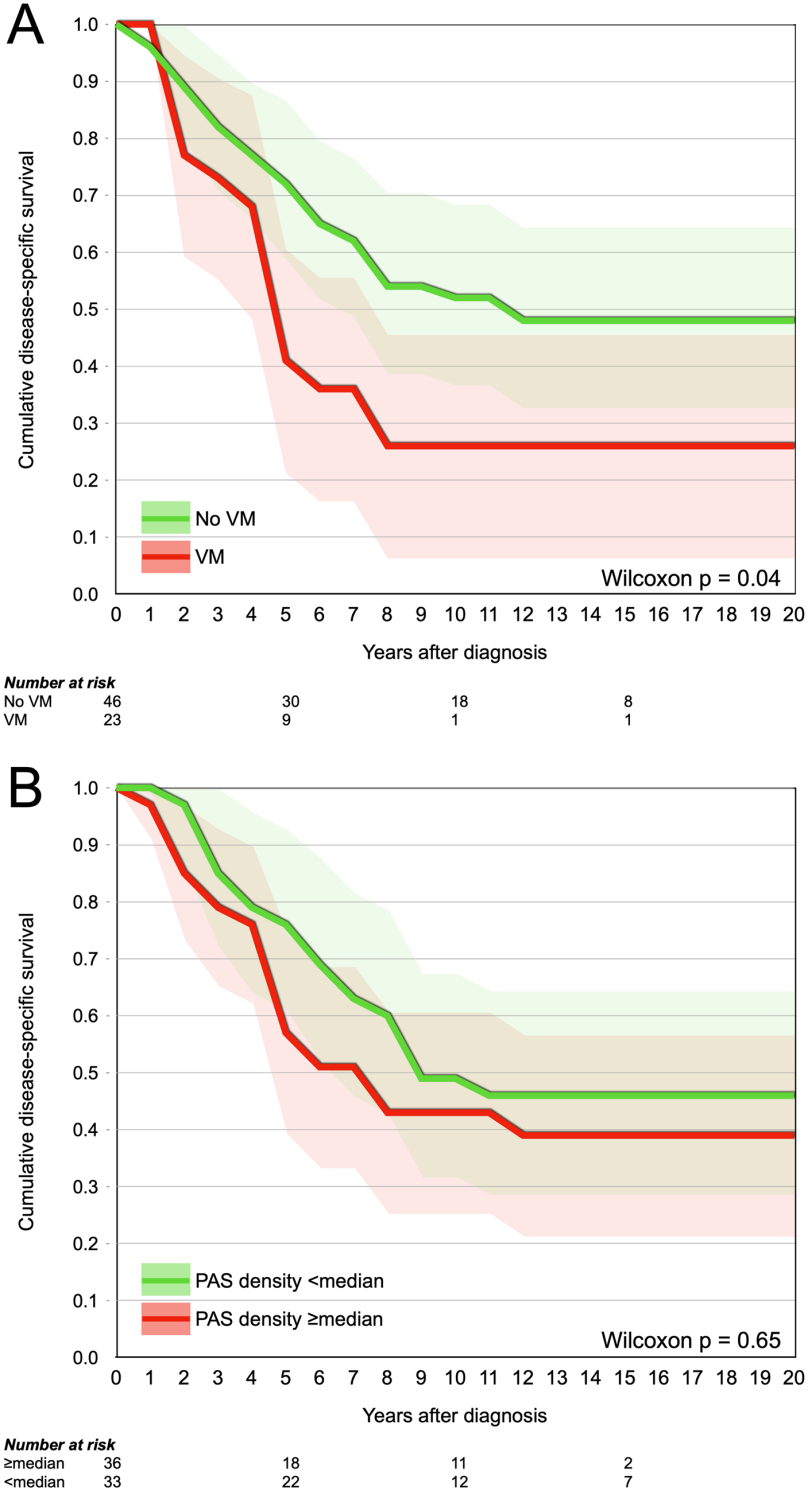
Cumulative disease-specific survival proportions. **A** Patients with presence of VM in their tumors (red) had significantly shorter disease-specific survival than patients without VM (green, Wilcoxon *p* = 0.04). **B** PAS density was not associated with disease-specific survival (**p** = 0.65). Colored areas represent 95% confidence intervals. *VM* vasculogenic mimicry

No presenting symptom (*p* = 0.54–0.75) or RD (*p* = 0.28) was associated with shortened disease-specific survival in the present cohort. The presence of VM was associated with significantly shorter disease-specific survival (*p* = 0.04, Fig. 5a), but not PAS density ≥ median (*p* = 0.65, Fig. 5b).

## Discussion

In this study, a shadow in the visual field as a presenting symptom of uveal melanoma, experienced by one in four patients, correlated strongly with patterns of VM and increased PAS density. Both of which are markers of aggressive disease (Foss et al. 1997a, b; Mäkitie et al. 1999; Kivelä et al. 2004; Stålhammar et al. 2019).

In 1999, Mäkitie et al. found a significant association between VM, microvascular density and exudative retinal detachment, independent of tumor size and ciliary body involvement (Mäkitie et al. 1999). Furthermore, when adjusting for the latter two factors, retinal detachment did not predict poor patient survival (Kivela et al. 2001). Consequently, we believe that there is no coincidence that we found a strong correlation between a shadow in the visual field, VM, and PAS density, and that in turn, this correlation may be the causal link between the shadow and poor prognosis. As we have shown previously, VM also correlates to gene expression class, expression of BAP-1, and macrophage infiltration (See et al. 2019; Stålhammar et al. 2019).

Gene expression profiles are of great importance in stratifying patient prognosis. Furthermore, mutations in the tumor suppressor BRCA1-associated protein-1 gene (*BAP1*), located on chromosome 3p, are mutated in a vast majority of metastasizing UM (Harbour et al. 2010; Karlsson et al. 2020). This mutation leads to a dedifferentiated stem-like phenotype that correlates strongly with poor prognosis (Harbour et al. 2010; Karlsson et al. 2020). *BAP1* is one of several genes that is associated with epithelial-to-mesenchymal transition (EMT) of the tumor cells. In this mutation sequence, the *BAP1* mutation has been assumed to occur relatively late, preceded by mutations in G-protein subunits including *GNA11* and *GNAQ* that are present in as high as 83–96% of UM (Decatur et al. 2016). Recently, however, we estimated that the *BAP1* mutation occurs when the primary tumor has a size of only a few malignant cells to 6 mm^3^ (Uner et al. 2021). Furthermore, other genetic aberrations may underlie the VM phenotype and increased PAS density. The role of non-coding RNAs has gained attention, including dysregulation of the possible oncogene *LINC00518* which may contribute to EMT, hypoxia-induced responses and thereby potentially vascularity and leakiness (Barbagallo et al. 2020).

PAS density should not be immediately translated into a representative of microvascular density, as a multitude of structures other than endothelial-lined blood vessels may be positively stained with PAS, including vasculogenic mimicry, macrophages, and glycogen stored in the cytoplasm of tumor and non-tumor cells (Foss et al. 1997a, b; Nowak et al. 1998; Meersseman et al. 2011). It is therefore important that the lack of prognostic significance of PAS density is not used to cast doubt on the prognostic significance of microvascular density.

The other presenting symptoms investigated by us are not associated with tumor vascularity or with patient outcome, neither in cumulative survival analysis nor in multivariate Cox regressions (Fili et al. 2021). Notably, blurred vision has been the most common symptom in our studies, with similar prevalence reported in a previous publication by Damato and Coupland (2012). Metamorphopsia, ocular pain and other symptoms were exceptions, each experienced by only 1 or 2% of patients.

There are several limitations to this study. The patients were included from one institution only and the data included were retrospective in nature, which limits our control over confounding factors. There is a range of other important prognostic factors in uveal melanoma that were not available for comparison in this study. Among these, analyses of the immunohistochemical expression of BAP-1 and P16INK4a, mutations in the *BAP1* gene, and gene expression classifications in relation to VM, PAS density, and presenting symptoms would have been highly interesting (Harbour et al. 2010; Onken et al. 2012; Russo et al. 2020; Herrspiegel et al. 2021). Our cohort was considerably smaller than our previous cohort in which the association between symptom and prognosis was found, which is the probable reason for the lack of survival differences between patients with and without specific symptoms herein, and for the non-significant difference in tumor diameters between tumors with and without VM. One-third of the included tumors had been previously treated with plaque brachytherapy, which may alter histological appearance. A previous case–control study has found a lower microvascular density in irradiated versus nonirradiated uveal melanomas (Toivonen et al. 2003). However, it cannot be excluded that this signifies a difference at baseline. We have no data on the growth rate of the tumors included, which may have offered alternative explanations to presenting symptoms and lack thereof. Furthermore, the description of a symptom is by definition based on a subjective experience and was not confirmed by additional testing, including perimetry. Some patients that reported a shadow may have experienced what others would describe as a floater or other, etc. Finally, even though any number of symptoms could be recorded by the patients in this study, it is possible that any symptom either is not noticed or not reported if it is obscured by other simultaneous symptoms. In that case, the true prevalence of symptoms would be higher than what we have reported here.

In conclusion, blurred vision is the most common presenting symptom in uveal melanoma. Metamorphopsia, pain, and other symptoms are all relatively rare. Patients presenting with a shadow have tumors with greater PAS density and are highly likely to have VM, likely explaining the previously reported association between this symptom and poor prognosis.

## Data Availability

The datasets used and/or analyzed during the current study are available from the corresponding author on reasonable request.
